# Time-dependent electrochemical characteristics of a phenolic and non-phenolic compound in the presence of laccase/ABTS system

**DOI:** 10.1371/journal.pone.0275338

**Published:** 2022-09-28

**Authors:** Rituparna Saha, Mainak Mukhopadhyay

**Affiliations:** 1 Department of Biotechnology, JIS University, Kolkata, West Bengal, India; 2 Department of Biochemistry, University of Calcutta, Kolkata, West Bengal, India; Babasaheb Bhimrao Ambedkar University, INDIA

## Abstract

The laccase/ABTS system has found several industrial applications ranging from biodeterioration to biodegradation and bioremediation. However, the capability of the laccase/ABTS system varies depending upon the type of substrate used. Voltammetric studies involving two widely used substrates, i.e., veratryl alcohol (VA) and alkali lignin (AL), were performed to gain new insight into the electrochemical behavior of the reactions. The individual electrochemical reactions established the differential nature of the two compounds over a concentration range, along with the mediator ABTS producing a distinguishing effect on their oxidative reactions, which was further studied over a 12hour period. It was followed by the reaction of both the compounds against the laccase/ABTS system that helped verify the role of the enzyme and the mediator in the electron transfer process and elucidate the mediated oxidations carried out by laccase against the phenolic and non-phenolic substrate through the process of cyclic voltammetry.

## Introduction

Laccase (EC 1.10.3.2) has become the most sought-after enzyme for various biotechnological applications in the industry [1]. Its biocatalytic role has been adopted across numerous industrial sectors, from textile, pharmaceutical, and food to cosmetic, paper, and pulp industries [2]. Extensive research into identifying its substrates and catalytic efficiency has made it possible to use the enzyme for bioremediation, biosensing technologies, and even biofuel cells [3]. The multi-copper oxidoreductase enzyme found widely distributed across the kingdom of prokaryotes and eukaryotes, and coupled with the generation of water at the expense of molecular oxygen, has a broad range of substrate specificity, which allows it to play a multitude of functions, including oxidation of phenols, detoxification and most importantly, degradation of recalcitrant compounds like lignin [4, 5].

However, one of the enzyme’s limitations is its low redox potential, making it unable to oxidize non-phenolic components, unlike its ligninolytic peroxidase counterparts [6]. The lignin polymer has both phenolic and non-phenolic fragments, with the non-phenolic moiety making up the most of it with a high redox potential [7]. An extensive study into fungal laccase has established the role of natural mediators like 3-hydroxyanthranilic acid (3-HAA), 4-hydroxybenzoic acid, and 4-hydroxybenzylic alcohol, which are synthesized as secondary metabolites, to increase the catalytic activity of laccase towards the non-phenolic moieties in recalcitrant compounds like lignin [8, 9]. These mediators act by shuttling electrons, which they achieve by oxidizing themselves with the enzyme’s help and producing stable radicals [10, 11]. These diffuse away from the enzyme pocket and enable the oxidation of the target compounds, either substrate of laccase but cannot interact with the enzyme due to its size and steric hindrances or due to their high redox potential [12, 13]. This process mimics natural and synthetic mediators in the laccase-mediator system (LMS) for industrial applications.

Out of the various mediators studied, the most commonly used synthetic mediator in LMS is 2,2’-azino-bis-(3-ethylbenzothiazoline-6-sulfonic acid) (ABTS). It has proven valuable and efficient in systems like oxidation of polycyclic aromatic compounds with laccases from different white-rot fungi [9, 14]. LMS, along with ABTS, has been developed as a good alternative for the delignification of raw materials in the paper and pulp industry compared to the various chemically induced bleaching treatments, which produce toxic sludge that is harmful to the environment [15, 16]. The laccase-ABTS system has been utilized to systematically bleach high-quality flax pulp without further chlorine treatment [17]. Other applications of the laccase/ABTS system include the pH-dependent decolorization and detoxification of dyes like Reactive Orange 1 and Reactive Red 198, released in textile wastewaters [18]. This unique system has also shown promise in reducing pesticide levels in water bodies and, as a result, reduces the environmental impact [19].

Irrespective of the many applications associated with LMS, there is still a lack of detailed understanding of laccase’s reaction mechanism and its mediator with a phenolic and a non-phenolic compound [20]. The most common techniques utilized for an LMS reaction involve conventional spectroscopy and analysis and characterization of the reaction products with GC-MS or HPLC [21, 22]. However, these processes take a prolonged time to achieve the desired results and therefore act as a hindrance to collectively associating each reactant’s functionality to the output of the reaction. To better understand the role of different types of mediators in the electron transfer process taking place between laccase and the phenolic and non-phenolic compound that is acting as the substrate, a more robust method is required to acknowledge and identify the redox potential and stability of the generated radicals in the LMS reactions [23]. This is where electrochemical analysis has found an important role. It has become easier to duplicate the reactions involving LMS and study their characteristics, including the reactive intermediates of various mediators [24]. In recent years, researchers have used cyclic voltammetry (CV) to estimate the differential effects of several LMS on a range of substrates [25]. These have helped rationalize the correlation between the mediator and its impact on substrates in the presence of laccase in the LMS reactions [26].

From a broad perspective, most of the substrates for laccase can be classified into either phenolic or non-phenolic compounds. Considering this, it is highly essential to decoding the influences that laccase and the various mediators have on these classes of compounds and how the LMS function in these reactions. For this study, we utilized CV to simulate LMS’s reactions and detect the behaviour of these electrochemical reactions by using a phenolic and a non-phenolic compound as a substrate. A more comprehensive account of the LMS mechanism could be interpreted by comparing and electrochemically analysing the electron transfer process in these two classes of compounds.

In the present work, we investigated the time-dependent change of a phenolic and a non-phenolic compound in the presence of the enzyme-mediator system. The electrochemical behavior and the electron transfer mechanism of the non-phenolic compound veratryl alcohol (VA) and the phenolic compound alkali lignin (AL) were analyzed in the presence of the mediator ABTS over a fixed concentration range. This was followed by the electrochemical reactions involving LMS of the two systems, i.e., VA and AL, set for a definite time. The comparison and change in the electrochemical characteristics and the electron transfer mechanism between the two sets of reactions could help to shed new light on the reaction mechanism of the LMS towards the two classes of substrates, which, in turn, could helpto facilitate the usage of the laccase-ABTS system in a variety of applications involving phenolic and non-phenolic compounds.

## Materials and methods

### Chemicals

All the chemicals used for the experiments in this study were of analytical grade and used without further purification. The enzyme laccase of the fungi *Trametes versicolor* and AL were bought from Sigma Aldrich, whereas ABTS and VA were obtained from SRL, India. Every component was solubilized and further diluted in sodium acetate buffer of strength 50mM (pH 5) prepared in deionized water from the Milli-Q system (Millipore, USA).

### Electrochemical analysis

Each of the reactions was prepared separately and conducted at a temperature of 25°C under stirring conditions to maintain environmental oxygen flow. For the time-dependent reactions, the individual sets were prepared and incubated for a set amount of time, after which they were also analyzed, maintaining the same conditions as the rest. In case of the non-phenolic compound i.e. VA–its individual reactions were carried out over a concentration range of 0.1-10mM, and the concentration of 2mM VA was selected for rest of the electrochemical reactions that was carried out. Likewise, in the case of the phenolic compound, AL–its individual reactions were carried out over a concentration range of 0.05-2mM, and similarly the concentration of 0.2mM was used for the rest of the electrochemical reactions. With respect to the synthetic redox mediator ABTS, a concentration range of 0.1-1mM was used for the reactions with VA and AL respectively. During the reactions that was performed for the time-dependent analysis the concentration of 0.5mM ABTS was used.

For the time-dependent analysis experiments carried out in relation to VA and AL, a total of five time-point stages were selected. The reactions were carried out at a time interval of 2 hours at first, with them being zero, 2 and 4hours respectively. Following the first three stages, the interval between was increased to 4hours–which are 8 and 12hours respectively. Each of the reactions was then analyzed through cyclic voltammetry.

The electrochemical experiments were carried out using a DY2113 Potentiostat (Digi-Ivy Inc.) with a single 25 ml cell comprising a standard three-electrode configuration—a platinum disk working electrode, an Ag/AgCl reference electrode, and a platinum wire counter electrode. The CVs for all the reactions were obtained under a fixed potential of +2 V to -2 V with a scan rate of 100 mV/s.

## Results and discussions

### Cyclic voltammetry of veratryl alcohol with laccase/ABTS system

The non-phenolic compound VA has long been identified as a secondary metabolite synthesized by lignin-degrading fungi [27]. Under controlled conditions, it has been found to play the dual role of a substrate and a mediator in ligninolytic enzyme systems [28, 29]. VA has been identified as an essential non-phenolic model compound used for a wide range of applications in the industry and catalyzed by laccase [30]. S1 Fig illustrates the CVs of VA recorded over a concentration range of 0.1-10mM. Compared to earlier reports, the concentration range was selected to observe the individual electrochemical behavior of VA in relation to concentration and reactivity [31]. As observed at the initial concentration of 0.1 mM, VA undergoes oxidation and reduction as indicated by the prominent anodic and cathodic peaks. This characteristic of VA undergoes relative changes as the concentration increases from 0.1mM to 0.5mM with changes in the oxidation rate, which, as observed, is directly proportional to the concentration change, with the anodic peak almost leveling out as a plateau with 10mM VA. In contrast to the oxidation rate, the reduction rate remains relatively the same throughout the concentration range. The change in oxidation and reduction indicates the formation of electroactive species that are oxidized and reduced continuously. The different concentration induces the generation of the electroactive species indicated by a high oxidation rate but gets reduced simultaneously, as observed from the CVs. This showed that the electroactive species formed during the reaction are highly stable and, therefore, have a consequential impact on the laccase/ABTS system.

Based on reaction characteristics and the use of VA in previous studies in its reaction with laccase, the concentration of 2mM VA was selected to undergo a series of electrochemical reactions against the synthetic redox mediator ABTS as shown in S2 Fig [32]. ABTS’s different concentrations vary depending on the oxidation and reduction rate of 2mM of VA. In relation to the oxidation rate of VA, the change in ABTS concentration does not produce any effect initially till 0.5mM. However, as the concentration increases to 0.8mM, a sub-anodic peak is observed that becomes merged with the initial anodic peak but is visible in the reaction with 0.8-1mM ABTS. Previous studies have reported that the mediator ABTS has a distinct electrochemical nature. It produces electroactive species, which includes mono- and di-cationic species indicative of multiple anodic and cathodic peaks that are observed as a result [16]. The sub-anodic peak observed in the higher concentrations of ABTS in S2 Fig specifies the formation of the same electroactive species. In the case of the earlier concentrations, the electroactive species of ABTS gets readily used by VA as ABTS helps the oxidation rate of VA electrochemically. This is similar to the analysis made in the previous studies, where it has been established that the dication of ABTS diffuses into the solution and drives the two-electron oxidation of VA to veratrylaldehyde, which further results in the regeneration of the cation radical [33]. In contrast, the higher concentrations of ABTS produce more of the electroactive species that do not get as readily utilized by VA; therefore, it appears as a sub-anodic peak in the CVs generated from the electrochemical reactions. ABTS has an entirely different effect on VA’s reduction capability compared to the oxidation rate. ABTS brings about a high reduction rate and keeps it uniform across all of its concentrations in reaction with 2mM of VA, as is evident by the cathodic peak compared to the CV of 2mM VA in S1 Fig. Therefore, the mediator ABTS helps to accelerate electron transfer towards the reduction part of VA that takes place in the cathode in comparison to the oxidation reaction, which takes place in the anode, providing the role that ABTS plays in the laccase/ABTS system with regards to a non-phenolic compound [34].

**Fig 1:**
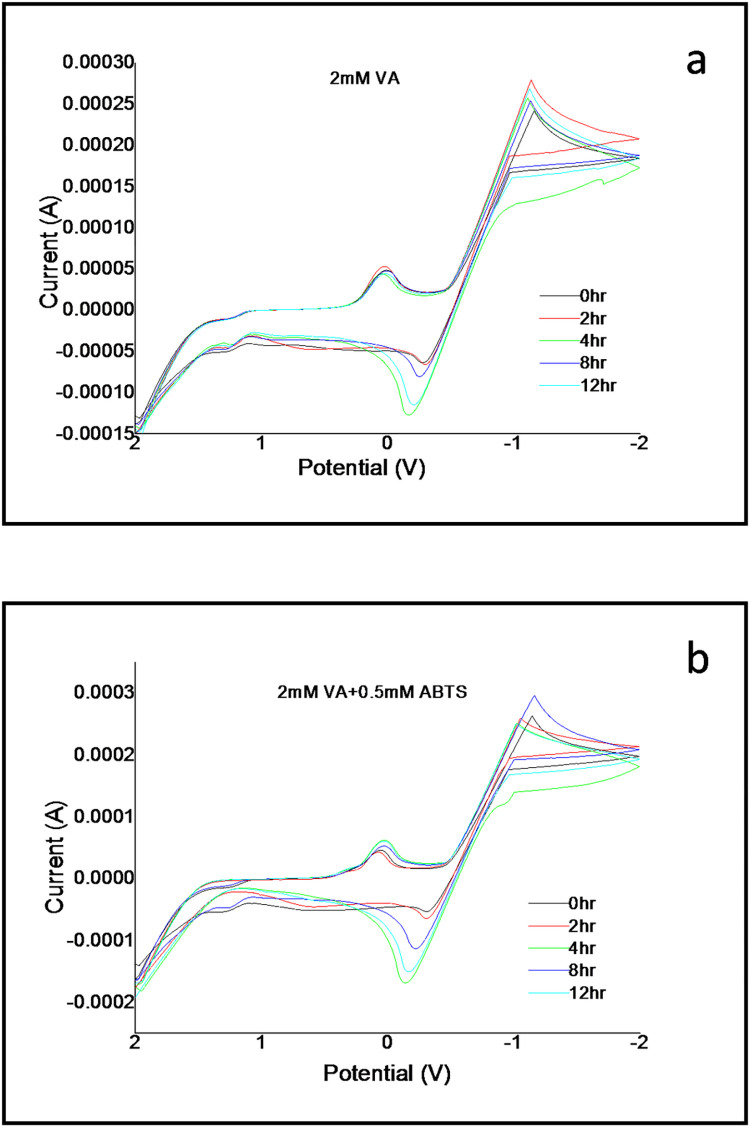
Cyclic voltammograms of VA (2mM) with the mediator ABTS (0.5mM) over 12 hours recorded in a buffered solution of pH 5 at a scan rate of 100mV/s.

The laccase/ABTS system is utilized in several industrial applications. The biological and chemical substrates that are involved and belong to the non-phenolic class of compounds take time to oxidize completely. A time-based analysis of the laccase/ABTS system components could help discern their electrochemical behavior as the reaction progresses. The electrochemical reaction of 2mM VA against 0.5mM ABTS was performed over period 12hours, as depicted in the CVs of Fig 1. As observed from the CV of 2mM VA in Fig 1A, the oxidation rate throughout the time interval remains the same as observed from the anodic peak. In contrast, the same reduction rate persists for 2hours. It decreases rather non-uniformly, implying a more extended period makes the electroactive species produced during oxidation quite unstable, making their reduction rate relatively slower than the reaction for 2hours. This is similar to the reaction of 2mM VA against 0.5mM ABTS (Fig 1B), as it shows the oxidation rate to be the same throughout 12hours, with the highest being at 2hours. Whereas in the case of reduction rate, it remains similar till 2hours after which the reduction rate reduces non-uniformly from 4-12hours. The non-uniform reduction rate is identical to that of Fig 1A, indicating the characteristics of the non-phenolic compound VA and not the mediator ABTS. In this case, the mediator oxidizes the substrate for 2hours. Its electroactive species fails to maintain the high reduction rate due to the unstable nature of the electroactive species produced by VA long-term.

**Fig 2:**
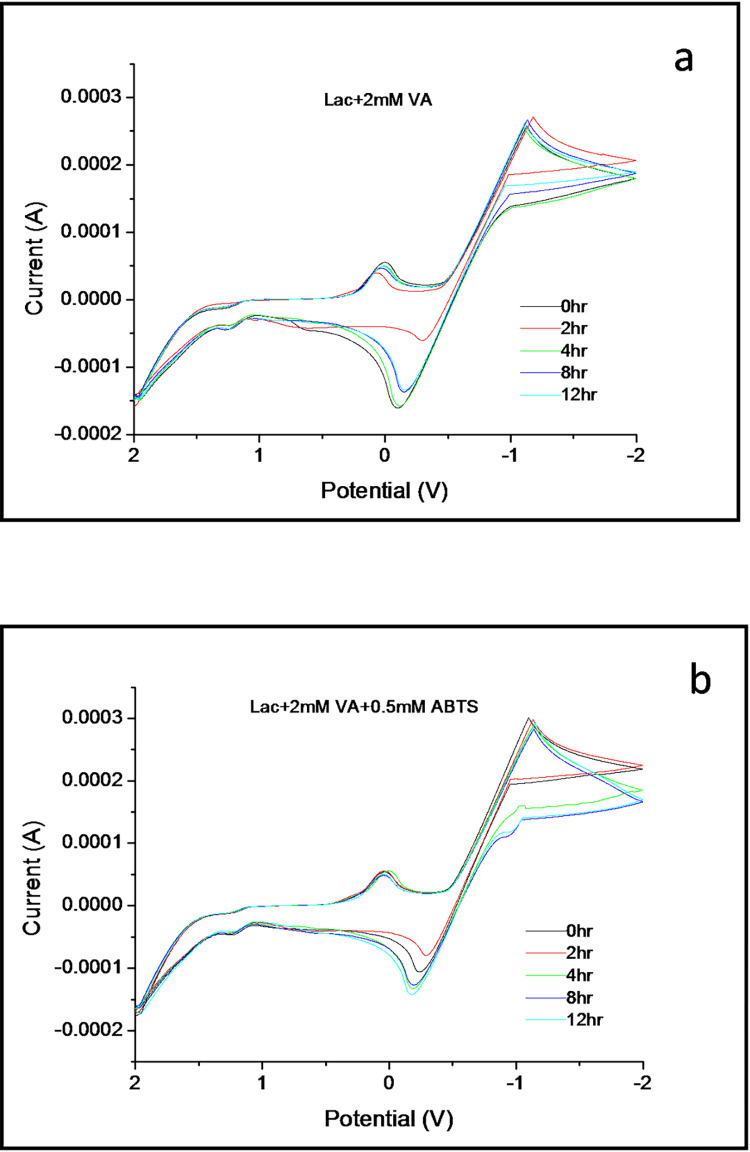
Cyclic voltammograms of VA (2mM) with *Trametes versicolor* laccase and the mediator ABTS (0.5mM) over 12 hours recorded in a buffered solution of pH 5 at a scan rate of 100mV/s.

The enzyme laccase brought a distinct change to the electrochemical reaction with VA in the presence of the mediator ABTS as evident from the CVs in Fig 2. During 12hours, VA gets the highest oxidation-reduction rate at 2hours in the presence of laccase (Fig 2A). Similar to the reactions in Fig 1, the enzyme’s oxidation rate is maintained at the same level throughout. But unlike the previous reactions, laccase induces the highest reduction rate at 2hours specifically. At zero hours, the reduction rate is reduced similar to the overall lreduction rate at 4-12hours. In comparison to ABTS, laccase takes a longer time to produce the same reduction rate as in Fig 1B, indicating that the electron transfer process from the enzyme’s active site to VA is slower than that of ABTS. This gets altered substantially in the reaction of VA in the presence of the laccase/ABTS system, as observed in Fig 2B. It is evident that the laccase/ABTS system profoundly affects the VA’s electrochemical reaction. Though the system still maintains the same oxidation rate throughout the 12hours, a stark change in the reduction rate occurs. The reduction rate is still the highest at 2hours, but, in this case, the reduction rate at zero hours is higher as compared to Fig 2A. Moreover, although the reduction rate gets non-uniformly reduced as the time increases, in the case of the laccase/ABTS system, it considerably remains elevated compared to the CVs in Figs 1 and 2A. This characteristic of the reaction highlights the mediator’s role in the shuttling of electrons between the substrate and the enzyme, which is similar to the reaction mechanism claimed in previous literature that laccase first oxidizes ABTS, after which the oxidized mediator converts non-phenolic substrates and proves to be consistent with the electron transfer mechanism of the laccase/ABTS system connecting the reactivity of substrates with their oxidation potential [35]. Additionally, electrochemical experiments have shown that the enzyme protects either one or both the electroactive species of ABTS as they are highly unstable. Therefore, the oxidized mediator remains in contact with the enzyme and does not diffuse freely in the solution. This paradox was clarified with the degradation by-products produced from the electroactive species of ABTS, which tends to have a much higher redox potential capable of oxidizing the non-phenolic substrates [36].

### Cyclic voltammetry of Alkali lignin with laccase/ABTS system

Lignin is a well-known recalcitrant polymer that forms an essential component in many agricultural and lignocellulosic biomass and other forestry residues. These different types of biomass are utilized in the industry for various applications [37]. The foremost issue involved with this type of biomass is the depolymerization of the lignin polymer. Pretreatment processes, including physical and chemical, have proved to be energy-intensive and economically expensive, along with the harmful effects the hazardous byproducts have on the soil and water ecosystem [38]. Biological approaches, including lignin-degrading fungi and the direct use of the laccase/ABTS system, has proved to be cost-effective with no requirement of additional energy and zero production of any harmful byproducts. The biological treatments involved bring about the depolymerization of lignin, but the detailed mechanism is still unknown [39, 40].

In the present study, we used AL–a phenolic polymer similar in structure to lignin to understand and gain insight into the compound’s electrochemical behavior in the presence of the laccase/ABTS system over a time span of 12hours. Previous studies and the compound’s molecular weight were considered to determine the concentration range of 0.05-2mM AL for the electrochemical reaction, as observed in S3 Fig [41]. Starting from the initial concentration of 0.05mM, AL gives a prominent anodic and cathodic peak representing the compound’s oxidation and reduction rate. This remains similar up to 0.2mM, after which a residual cathodic peak start forming in correlation with the cathodic peak from the concentration of 0.4mM, but possessing a high reduction rate. In the reaction of 2mM AL, multiple anodic and cathodic peaks are formed, with a high oxidation and reduction rate, respectively. AL is a highly complex polymer, and its electrochemical reaction gives rise to multiple electroactive species, which gets depicted through the formation of multiple anodic and cathodic peaks. But this specific characteristic is concentration-dependent, which signifies that the electron transfer process between the primary electroactive species is responsible for inducing the formation of secondary electroactive species. This can serve to indicate the role that the electrochemical nature in correlation with AL’s concentration plays a significant role in the presence of the laccase/ABTS system.

In association with previous reports, 0.2mM AL was used to react against a varied concentration range (0.1-1mM) of the synthetic redox mediator ABTS as evident from S4 Fig [42]. Right from the initial concentration, although ABTS doesn’t have any outright effect on the standard oxidation-reduction rate of AL, it initiates the production of the secondary cathodic peak with a high reduction rate similar to that observed in the higher concentrations of AL in S3 Fig. With the increase in the mediator’s concentration, the cathodic peak becomes more prominent and distinguishable from the primary cathodic peak. In comparison to the reaction of 2mM VA with ABTS in S2 Fig, the mediator in AL also affects the reduction rate producing the secondary cathodic peak. Still, unlike the reaction with VA, no secondary anodic peak referring to ABTS’s electroactive species was visible in any electrochemical reactions in S4 Fig, whether in the lower or higher concentrations. It might be because AL readily uses up the electroactive species formed by ABTS because of its complex chemical structure. Another interesting observation that can be accounted from the CVs in S4 Fig is that the rapid electron transfer process of ABTS leads to the formation of the secondary cathodic peak, pointing out that the mediator accomplishes similar electrochemical behavior of AL at lower concentrations in comparison to AL as observed in S3 Fig.

**Fig 3:**
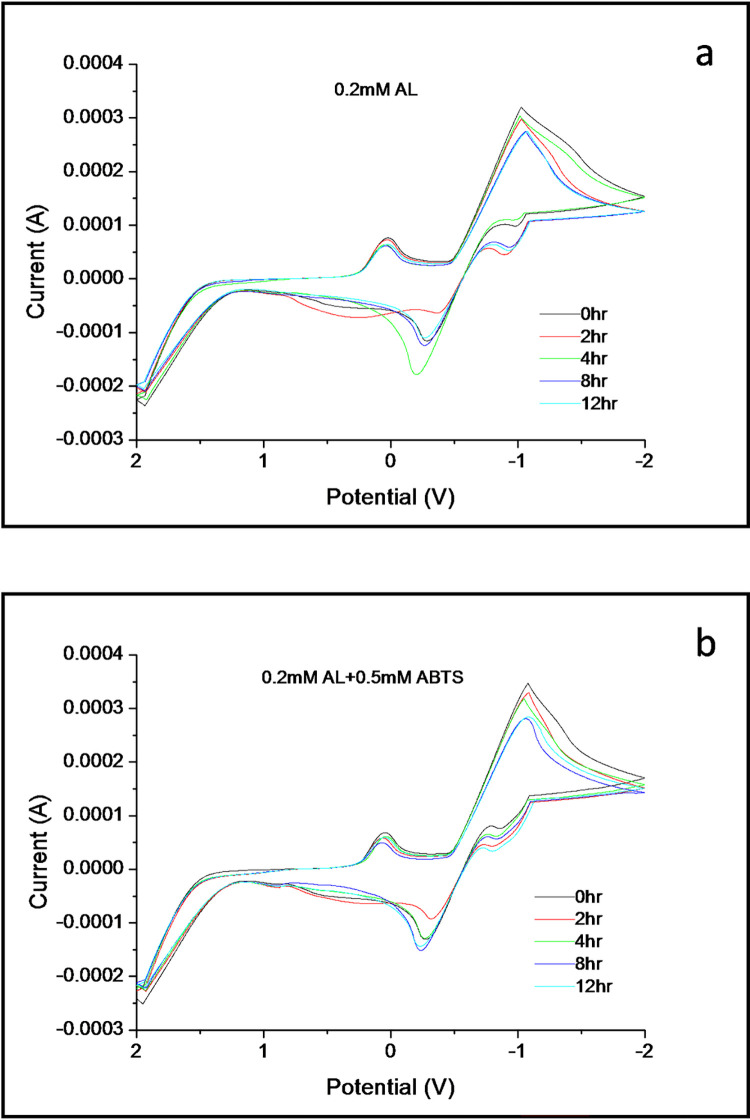
Cyclic voltammograms of AL (0.2mM) with the mediator ABTS (0.5mM) over 12 hours recorded in a buffered solution of pH 5 at a scan rate 100mV/s.

In the case of the reactions concerning 0.2mM AL against the mediator ABTS over a time span of 12hours as depicted in Fig 3, AL, similar to VA, maintains the oxidation rate at the same level throughout the entire time period. In contrast, the reduction rate is the highest at 2hours and is higher than that at zero hours (Fig 3A). After this, the reduction rate becomes inconsistent as time moves from 4-12hours. One distinguishing feature of the reaction of AL is the presence of the secondary cathodic peak, which is hardly visible at zero hours but becomes prominent as the time period increases. Although unstable, the secondary cathodic peak has a higher reduction rate than the primary one. Similar to the reduction rate, the secondary peak achieves at 8-12hours, unlike the primary one. This characteristic of the secondary peak gets altered extensively during the VA’s reaction with the mediator ABTS in Fig 3B. The mediator, along with maintaining the same oxidation-reduction rate, also generates a prominent and more significant secondary cathodic peak that, compared to the one developed in Fig 3A, has a much more stable reduction rate throughout the entire period. This behavior aptly justifies the role ABTS plays in the electron transfer process of AL’s electrochemical reaction. The mediator elevates the shuttling of electrons between the electroactive species produced by AL and remarkably induces the swift formation of the secondary cathodic peak. As a result, the mediator has different outcomes against substrates of different chemical structures and a paramount role in the laccase/ABTS system.

**Fig 4:**
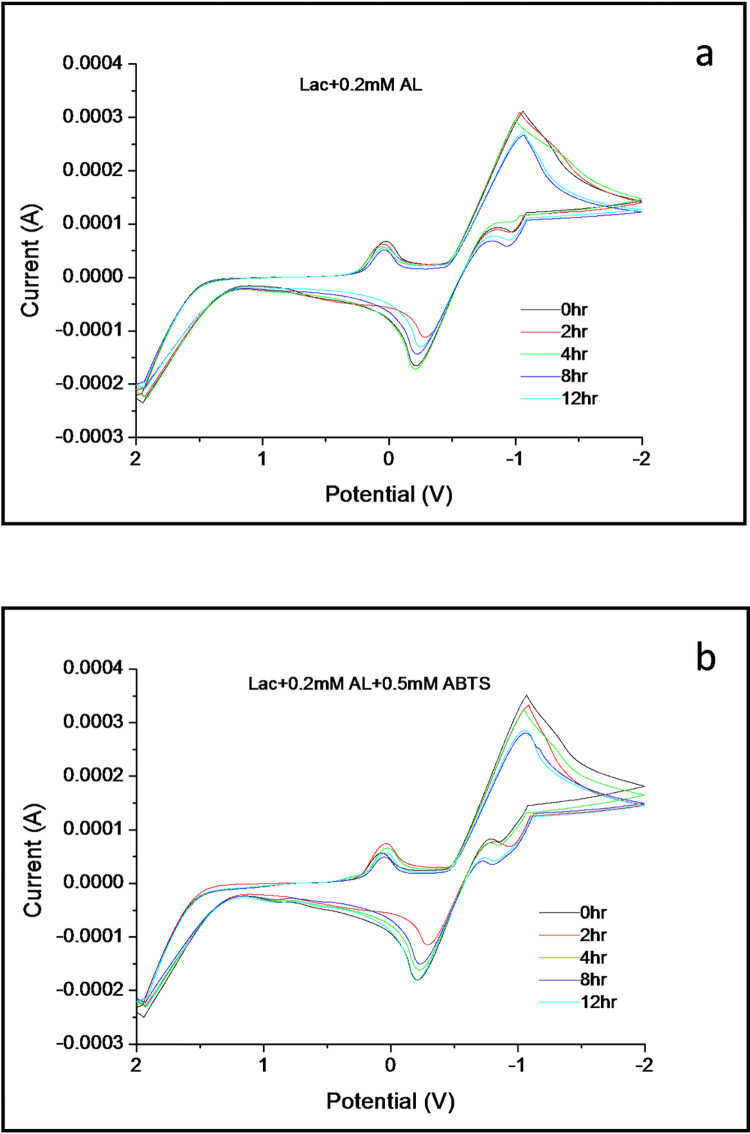
Cyclic voltammograms of AL (0.2mM) with *Trametes versicolor* laccase and the mediator ABTS (0.5mM) over 12 hours recorded in a buffered solution of pH 5 at a scan rate of 100mV/s.

In AL’s electrochemical reaction with the enzyme laccase, as observed from the CV in Fig 4A, the electrochemical effect of the enzyme is quite different from that of the mediator and closely similar to that of AL in Fig 3A. The enzyme maintains the same oxidation rate of AL along with leveling out the reduction rate throughout the entire time span. This contrasts with the reduction rate observed in the CVs in Fig 3, although the reduction rate is highest at 2hours. The electron transfer rate from the active site to AL plays a massive role in stabilizing and maintaining the regular reduction rate in the electrochemical reaction. The secondary cathodic peak is another distinguishing feature in AL’s reaction with laccase (Fig 4A). Although similar to the one in Fig 3A, it is highly stable and almost correlates with the primary cathodic peak. It is evident from the CV in Fig 4A that the lower reduction rate of the primary cathodic peak is closely related to the formation of the secondary cathodic peak. This highlights that laccase, while being responsible for the electron transfer to and from AL, also induces the formation of secondary electroactive species when the reduction rate increases, providing insight into a distinguishing characteristic of the enzyme when responding to different varieties of substrates [43]. But, when the complete laccase/ABTS gets involved in AL’s reaction as depicted in the CV of Fig 4B, the voltammogram has the characteristic similar to the CV of Fig 3B. Starting from the similar oxidation-reduction rate throughout the entire time period, the CV also exhibited the secondary cathodic peak with the same characteristics, implicating that in the case of the laccase/ABTS system, it’s the mediator that drives the electron transfer process in the reaction with AL.

Comparing the electrochemical characteristics of the laccase/ABTS with the non-phenolic compound VA and the phenolic compound AL, it was found that the two classes of compounds possess different types of electrochemical behavior. This was consistent with the mediator ABTS, which produced differential effects regarding the two compounds, implying the mediator’s response corresponds to the types of substrates used for the reaction. Although the impact of both the enzyme and the mediator was less pronounced on the two compounds’ oxidation rate, they had a distinctly different effect towards the reduction rate and efficiency of maintaining it throughout the 12hour time period. Therefore, the present study confirmed that cyclic voltammetry could be a valuable tool in understanding the time-dependent influence of the laccase/ABTS system on the oxidation-reduction and the overall electrochemical behavior of different groups of substrates. Besides, this could help understand the reaction mechanism of the laccase/ABTS system and could also broaden the applicability of the system.

## Conclusions

In this study, the electrochemical behavior of the laccase/ABTS system towards a phenolic and non-phenolic substrate was analyzed through cyclic voltammetry. Reactions were carried out over 12hours to gain insight into the electron transfer mechanism involving the two substrates. In the presence of the mediator ABTS, the substrates presented highly distinguishable effects related to their oxidative capacity. However, despite the mediated oxidations catalyzed by ABTS, there is relative ambiguity regarding a detailed understanding of the complex reaction mechanisms involved with the interactions that takes place between the enzyme and the substrates in the presence of the mediator. Although the laccase/ABTS system has a wide range of applicability in industrial processes, its use is restricted due to the limited evidence available about the reaction mechanisms that operate in the oxidation reactions. Further studies regarding the reaction intermediates and mechanisms involved are therefore necessary.

## Supporting information

S1 Fig Cyclic voltammograms of VA (0.1-10mM) in a buffered solution of pH 5 recorded at a scan rate of 100mV/s. (TIF)

S2 Fig Cyclic voltammograms of VA (2mM) with the mediator ABTS (0.1-1mM) in a buffered solution of pH 5 recorded at a scan rate of 100mV/s. (TIF)

S3 Fig Cyclic voltammograms of AL (0.05-2mM) in a buffered solution of pH 5 recorded at a scan rate of 100mV/s. (TIF)

S4 Fig Cyclic voltammograms of AL (0.2mM) with the mediator ABTS (0.1-1mM) in a buffered solution of pH 5 recorded at a scan rate of 100mV/s. (TIF)

S5 Fig Cyclic voltammograms of Guaiacol (0.1–10 mM) in a buffered solution of pH 5 recorded at a scan rate of 100 mV/s. (TIF)

S6 Fig Cyclic voltammograms of Guaiacol (2 mM) with the mediator ABTS (0.1–1 mM) in a buffered solution of pH 5 recorded at a scan rate of 100 mV/s. (TIF)

S7 Fig Cyclic voltammograms of Guaiacol (2 mM) with the mediator ABTS (0.5 mM) over 12 hours recorded in a buffered solution of pH 5 at a scan rate of 100 mV/s. (TIF)

S8 Fig Cyclic voltammograms of Guaiacol (2 mM) with *Trametes versicolor* laccase and the mediator ABTS (0.5 mM) over 12 hours recorded in a buffered solution of pH 5 at a scan rate of 100 mV/s. (TIF)

S1 File Cyclic voltammetry of Guaiacol with laccase/ABTS system. (DOCX)
