# Supplementary figures and images for: Time-dependent electrochemical characteristics of a phenolic and non-phenolic compound in the presence of laccase/ABTS system
Source: PLoS One. 2022 Sep 28;17(9):e0275338. doi: 10.1371/journal.pone.0275338 (PMC9518846; doi:10.1371/journal.pone.0275338)

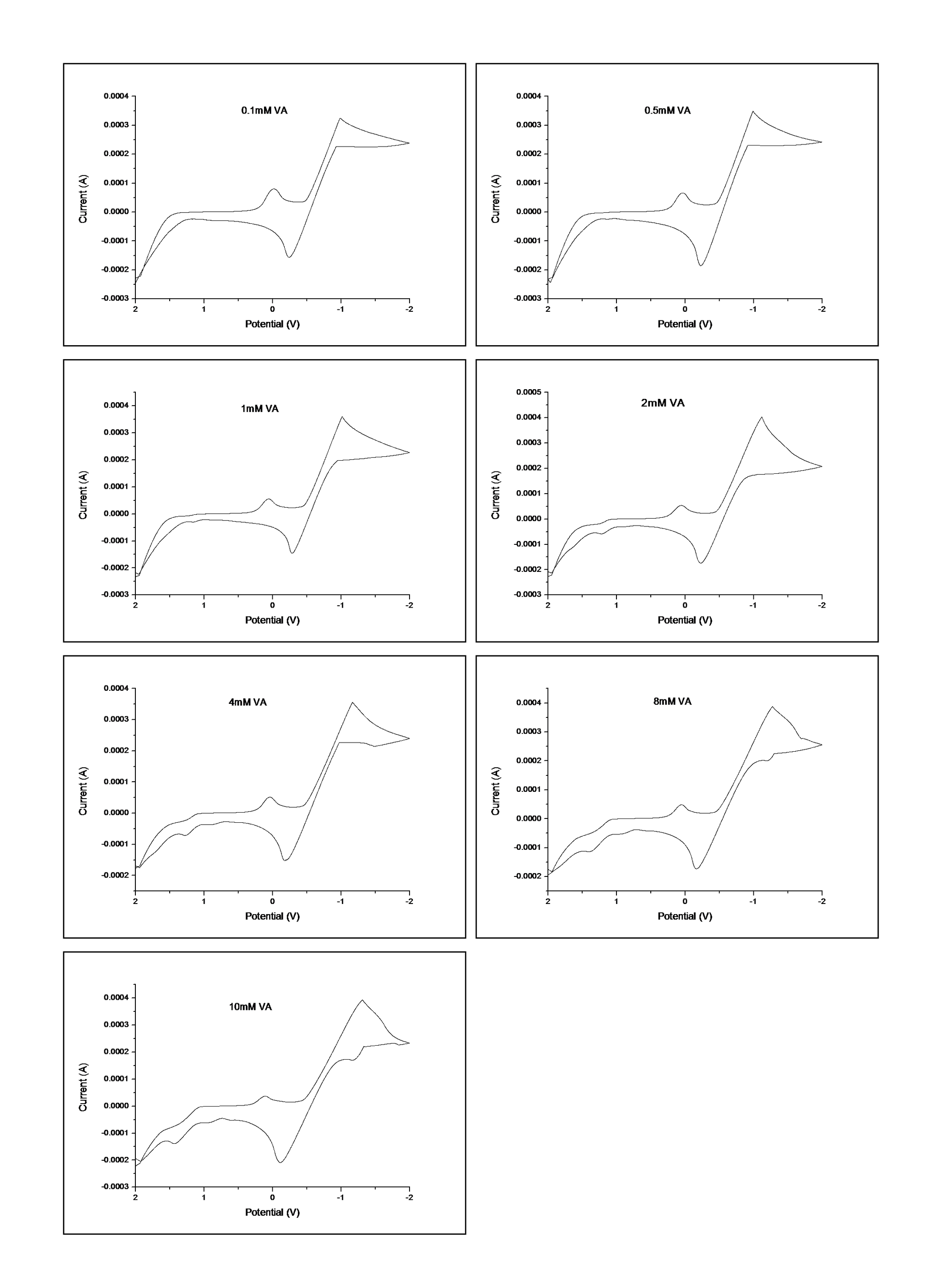

Supplement: S1 Fig — (TIF) [file pone.0275338.s001.tif]

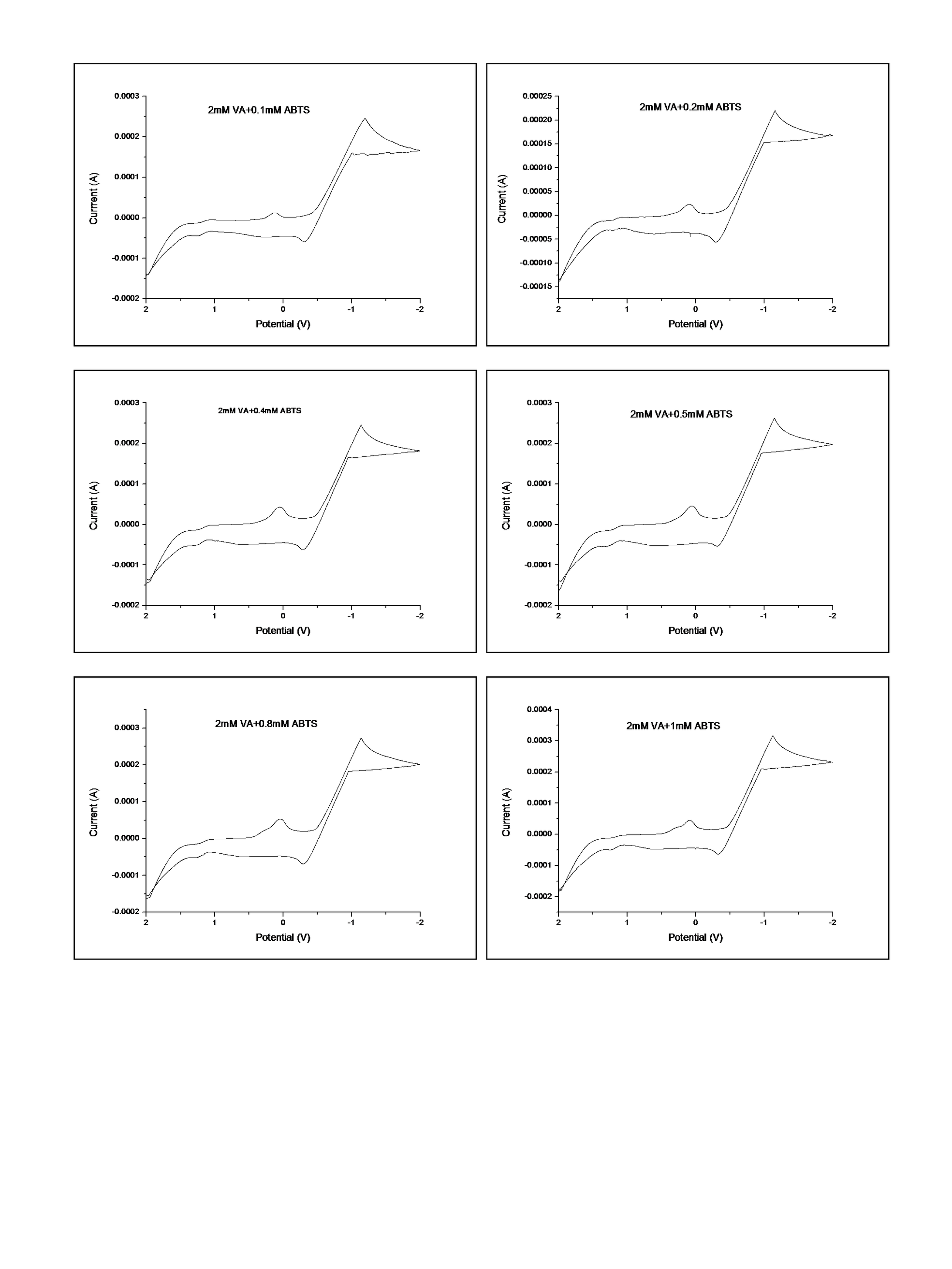

Supplement: S2 Fig — (TIF) [file pone.0275338.s002.tif]

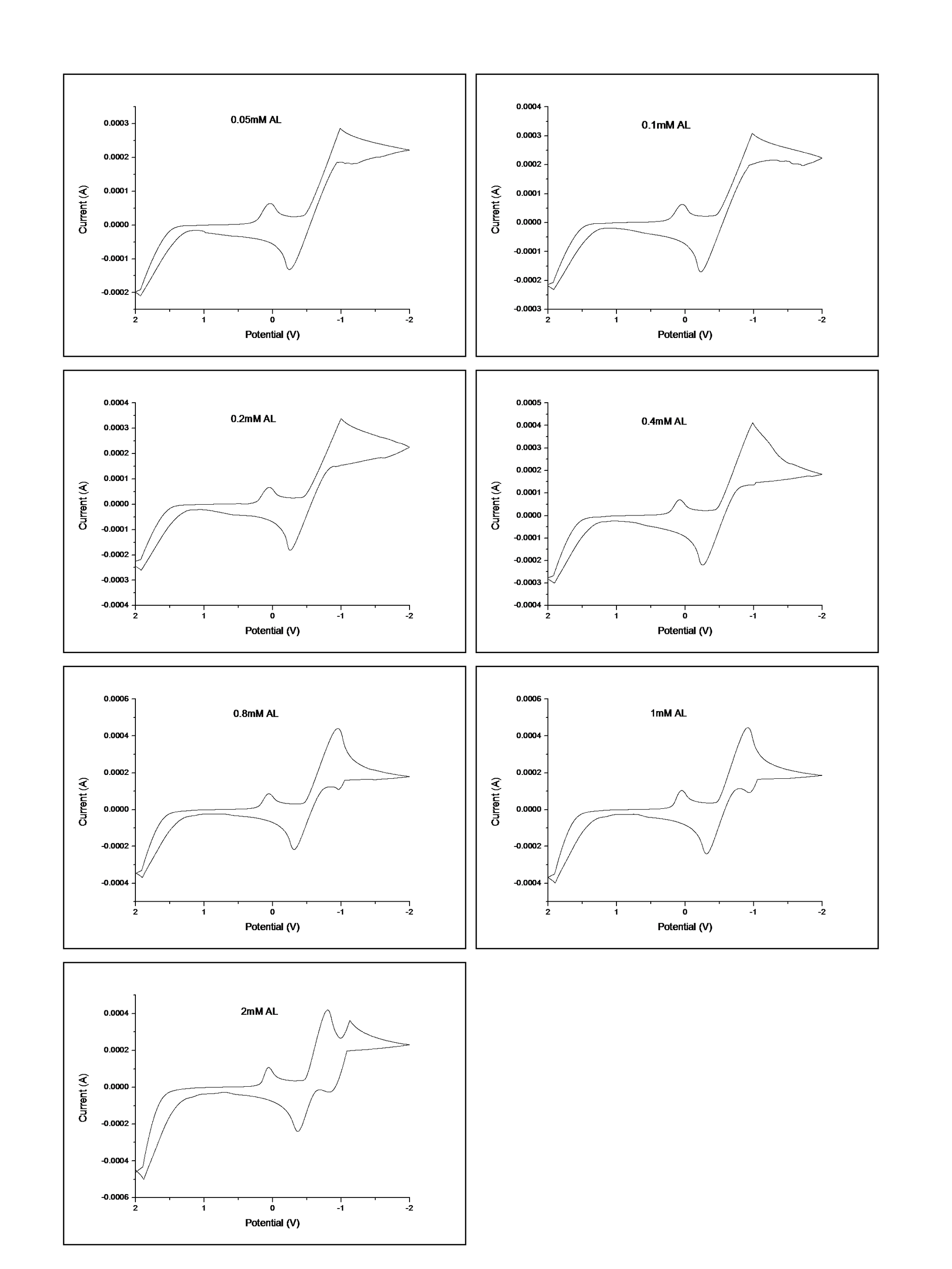

Supplement: S3 Fig — (TIF) [file pone.0275338.s003.tif]

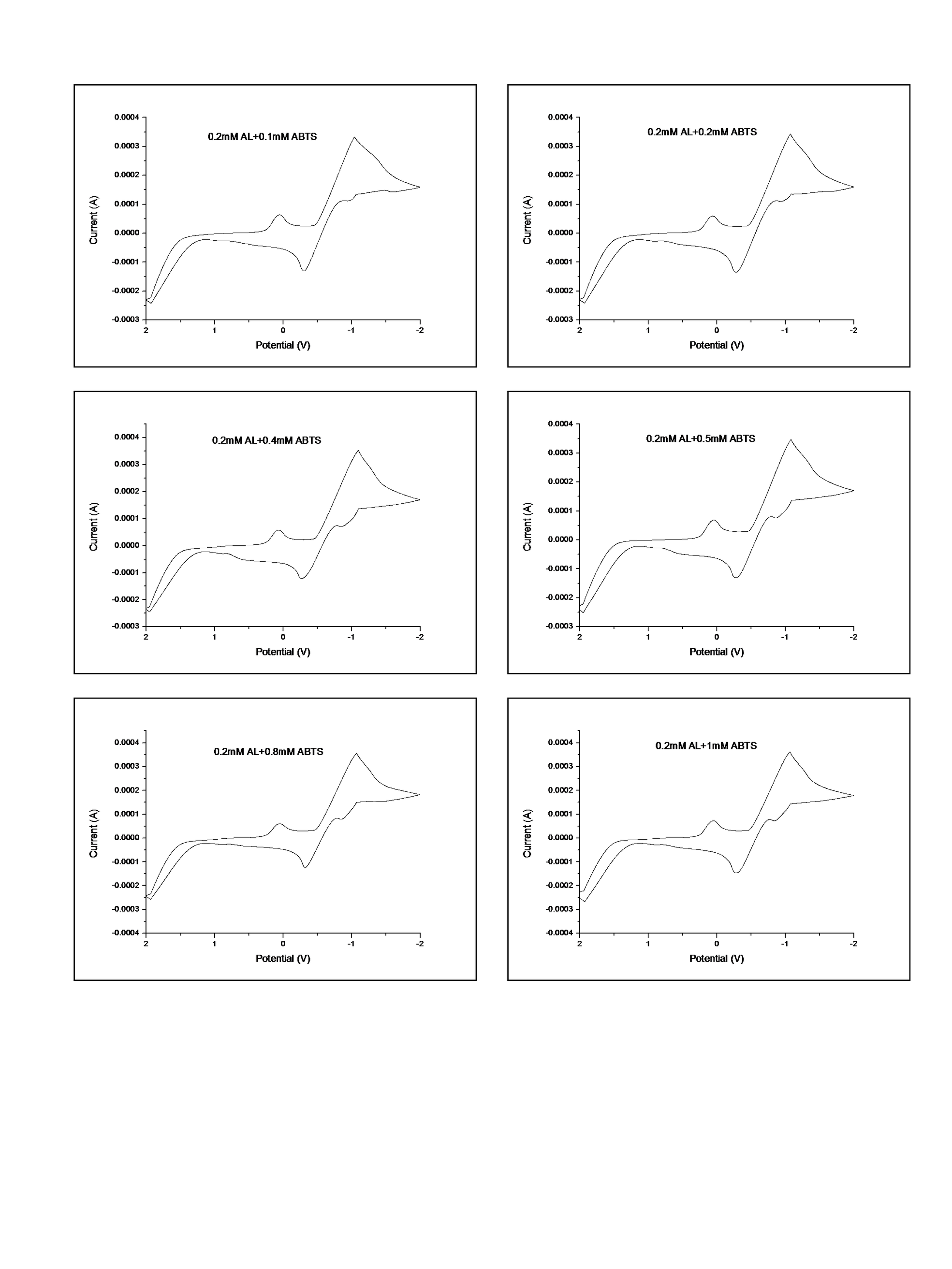

Supplement: S4 Fig — (TIF) [file pone.0275338.s004.tif]

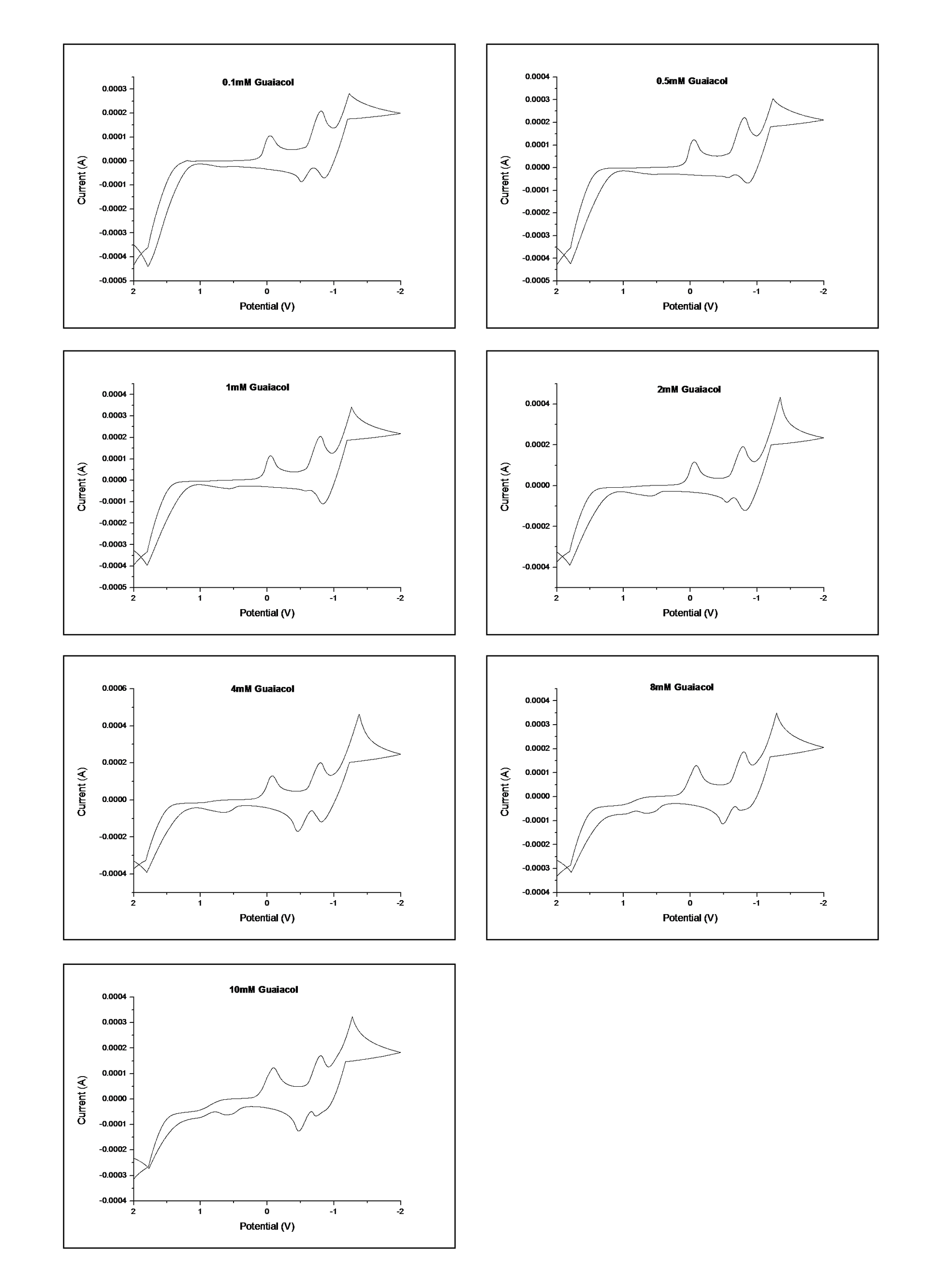

Supplement: S5 Fig — (TIF) [file pone.0275338.s005.tif]

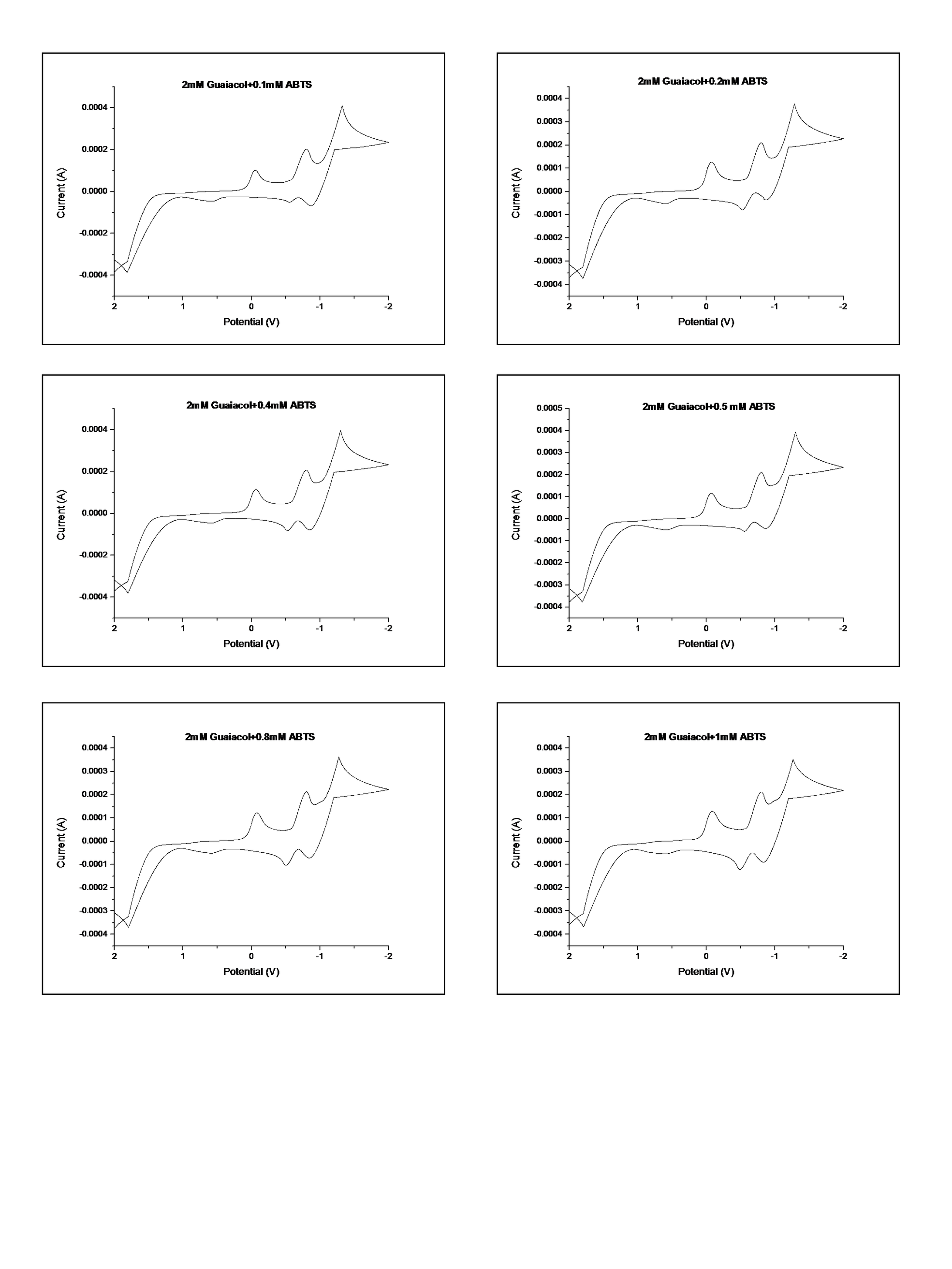

Supplement: S6 Fig — (TIF) [file pone.0275338.s006.tif]

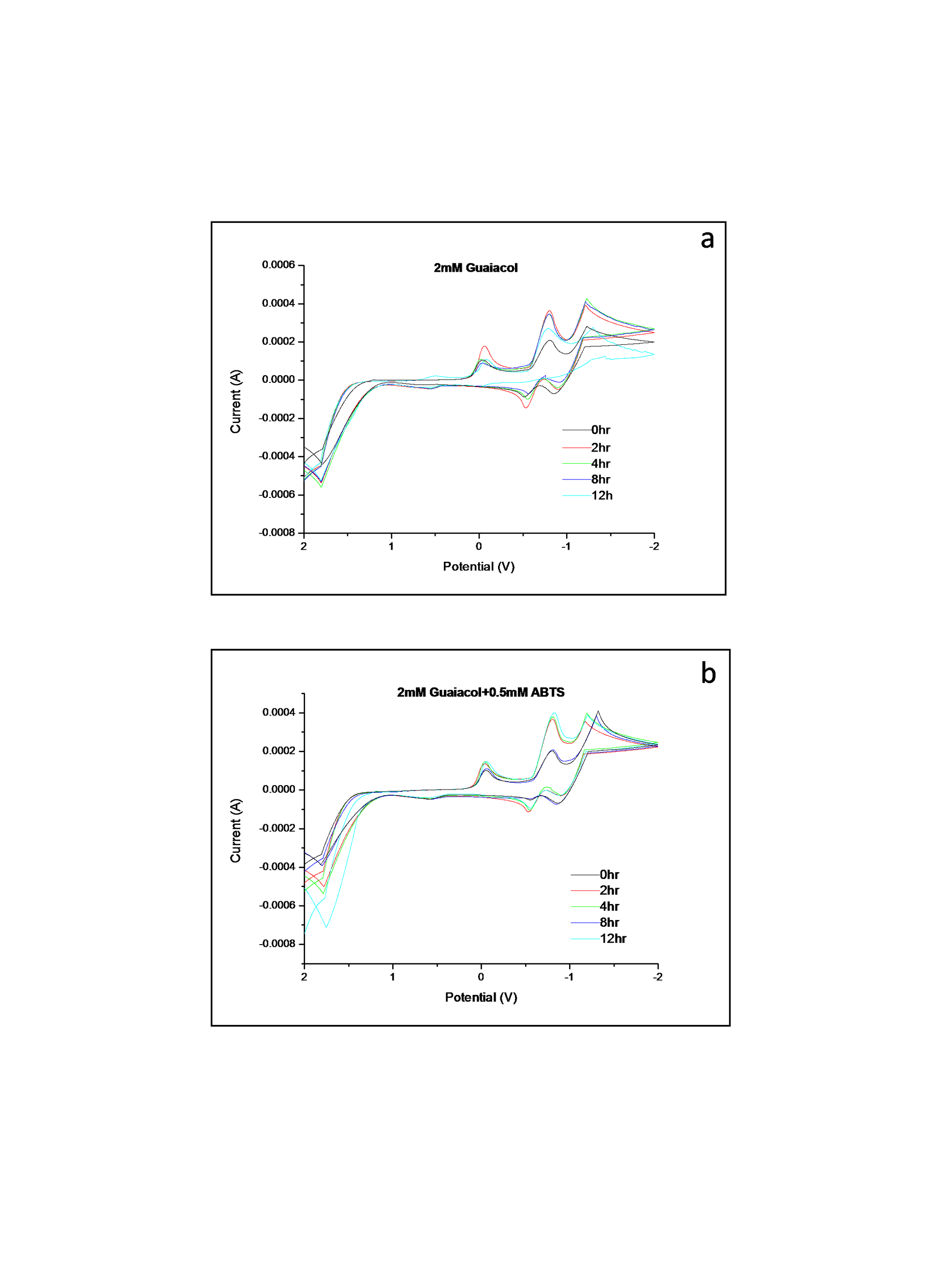

Supplement: S7 Fig — (TIF) [file pone.0275338.s007.tif]

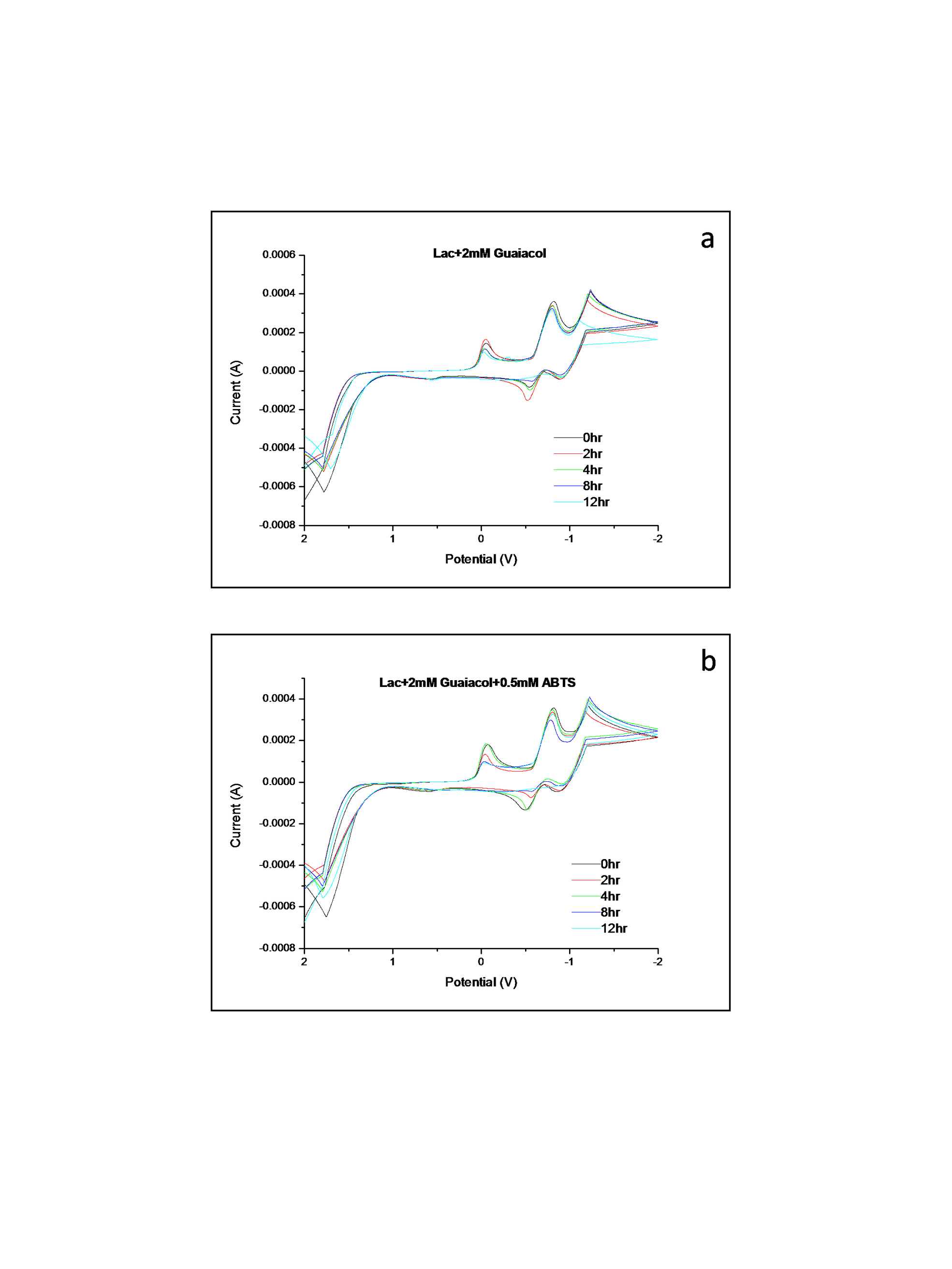

Supplement: S8 Fig — (TIF) [file pone.0275338.s008.tif]
