# Supplementary material for: Time-dependent electrochemical characteristics of a phenolic and non-phenolic compound in the presence of laccase/ABTS system
Source: PLoS One. 2022 Sep 28;17(9):e0275338. doi: 10.1371/journal.pone.0275338 (PMC9518846; doi:10.1371/journal.pone.0275338)
Supplement: S1 File — (DOCX) [file pone.0275338.s009.docx]

**Supplementary Text**

Although AL has been used as the phenolic substrate, the compound itself is a very complex polymer that contains various structures (such as the remaining active secondary –OH groups) and impurities (such as sulfur) which have the probability of affecting the electrochemical behavior of the reactions performed. Therefore, an additional simple model phenolic compound, i.e., Guaiacol, has also been used to observe and compare the electrochemical behavior to the non-phenolic substrate, VA.

**Materials and methods**

**Electrochemical analysis**

Similar conditions were maintained for each of the reactions performed with Guaiacol. Individual reactions were carried out over a concentration range of 0.1-10mM, and the concentration of 2mM was then selected for the rest of the electrochemical reactions. In the case of ABTS, a concentration range of 0.1-1mM was used, followed by the utilization of the 0.5mM concentration for the time-dependent analysis. The time-dependent reactions were similarly carried out at five time-point stages, with the analysis obtained through cyclic voltammetry.

**Results and discussions**

**Cyclic voltammetry of Guaiacol with laccase/ABTS system**

Guaiacol was selected as a lignin model compound to study the oxidation rates catalyzed by the laccase/ABTS system. It represents one of the most critical structural patterns found in lignin. The individual electrochemical behavior of guaiacol was recorded over a concentration range of 0.1-10mM, as shown in S5 Fig. Starting from the initial concentration, the CVs were characterized by two anodic peaks similar to earlier findings [43]. The first peak corresponds to the oxidation of the phenol group to a phenoxy radical, and the second peak to the oxidation of the aromatic ring to an aryl cation. Two cathodic reduction peaks followed the anodic peaks. However, compared to the much stable anodic peaks, the cathodic peaks were unstable and tended to shift in their cathodic peak potentials with variation in concentration. The lower concentrations were marked by a single cathodic peak which started changing its peak potential and shifting towards a second cathodic peak starting from 2mM. At 4mM, two cathodic reduction peaks were present, soon becoming one as the concentration changed from 8mM onwards to 10mM. Although anodic peaks provide sufficient information on the redox properties of the lignin model compounds, the change in the cathodic peaks as a result of concentration difference makes it highly imperative to acknowledge the resulting differences present in the reduction phase. As a result, the 2mM concentration of guaiacol was further selected for utilization in the rest of the electrochemical reactions.

In the case of the reaction with the mediator ABTS (S6 Fig), guaiacol maintained both the anodic peaks as observed previously. However, the different concentrations of ABTS produced a varied effect on the cathodic peaks. Previous findings have implicated that ABTS itself produces two electroactive species that give off multiple anodic and cathodic peaks. Against guaiacol, however, the peak potentials of the anodic peaks remain similar as in S5 Fig, but with an increase in ABTS concentration, two prominent cathodic peaks were observed. Starting with the initial concentration of 0.1mM of ABTS, a single cathodic peak was observed alongside a highly insignificant second cathodic peak. However, as the concentration increased from 0.2mM onwards, the second cathodic peak slowly became prominent besides the first peak. 0.5mM ABTS had the highest peak potential of the cathodic peaks, decreasing as the concentration increased from 0.8 to 1mM of ABTS. In contrast with S5 Fig, the cathodic peaks in S6 Fig were not only stable but also remained prominent throughout the reactions. There was no shifting or instability observed in the reduction peaks of guaiacol in the presence of ABTS. This change indicates that the electroactive species of ABTS can stabilize the reduction process of guaiacol through proper electron transfer between the two compounds.

The time-based analysis of guaiacol against the laccase/ABTS system was performed to study the electrochemical behavior as depicted in the CVs in S7 Fig. As depicted in S7a Fig, the oxidation rate of the anodic peaks of 2mM guaiacol differs over the 12hours. The first anodic peak has the highest oxidation rate at 2hours and then decreases to remain at the same rate over the rest of the time interval. In contrast, the second anodic peak displays a different feature altogether. The oxidation rate of the second anodic peak reaches its highest at 2hours and remains at the same potential till 8 hours and then decreases at 12hours but does not reach the initial stage of zero hours, unlike that of the first anodic peak. In the case of the two cathodic peaks, the reduction rate remains the same over the entire time interval and increases the highest at 12hours. The first anodic peak exhibits the same oxidation rate as the reaction against ABTS in S7b Fig. In contrast, the second anodic peak establishes the highest oxidation rate from 2-4hours. It then decreases to return to the same rate at the initial phases, indicating ABTS plays a vital role in increasing the oxidation rate of the phenol group to its phenoxy radical, which reaches equilibrium by the end of 4hours. However, the reduction rate develops into two separate cathodic peaks within 4hours, with ABTS stabilizing the electron transfer rate between the oxidation and reduction phases of guaiacol over the short time interval.

Laccase brought a distinct change to the oxidation rates of guaiacol over the 12hours, as observed in S8 Fig. As far as the oxidation rates are concerned in S8a Fig, the enzyme keeps the potential of both the anodic peaks almost uniform throughout the 12 hours time interval compared to the CVs in S7 Fig. Contrary to ABTS, laccase stabilizes the oxidation rate of the second anodic peak due to guaiacol interacting with the active site of the enzyme. Nevertheless, the reduction rate in the presence of laccase has no distinct changes as detected in the cathodic peaks of S8a Fig. The cathodic peaks have the same features as guaiacol individually, with the highest reduction rates at 12hours. This is in sharp contrast to VA, as seen in Fig 2, where the reduction rates were reduced over time due to the slow electron transfer process from the active site to VA. In contrast, the reduction rates of guaiacol increase substantially throughout the period and reach their highest at 12hours. This establishes that the electron transfer rates between the active site and guaiacol were more stable and uniform for an extended period than its non-phenolic substrate. The electrochemical behavior of guaiacol in the presence of the laccase/ABTS system in S8b Fig exhibited similar characteristics to that of S8a Fig. In the reaction studied in S8b Fig, uniform oxidation rates were observed in the anodic peaks from the initial phases, which gradually declined as they reached the 12hours interval. The reduction rates were observed as two separate cathodic peaks starting from zero hours onwards, which ultimately increased from 8-12hours intervals with no reduction peak observed towards the end of the period. This aspect of the reaction highlights that the electrochemical activity of ABTS gets preceded by laccase where guaiacol is concerned, and the electron transfers were most overwhelmingly taken over by the enzyme, which stays stable for an extended period as far as guaiacol is concerned but reduces proportionately after 8hours. In addition, it can also be discerned that the enzyme loses its oxidative capacity, as evident from the sharp decline in the oxidation rates of both the anodic peaks over time.
